# Asymmetric impact of oil prices on stock returns in Shanghai stock exchange: Evidence from asymmetric ARDL model

**DOI:** 10.1371/journal.pone.0218289

**Published:** 2019-06-18

**Authors:** Muhammad Kamran Khan, Jian-Zhou Teng, Muhammad Imran Khan

**Affiliations:** School of Economics, Northeast Normal University, Changchun, Jilin, China; The Bucharest University of Economic Studies, ROMANIA

## Abstract

This study scrutinized the asymmetric impact of oil prices on stock returns in Shanghai stock exchange with data (January 2000 to December 2018) by using asymmetric ARDL model. The examined results of asymmetric autoregressive distributed lag model indicate that cointegration exists between the oil prices and the stock returns. Results of asymmetric autoregressive distributed lag model confirm that both in the long run and the short run increase in oil prices have a negative impact on the stock returns of Shanghai stock exchange while decrease in the oil prices has a positive impact on the stock returns. The examined results of this study recommend that oil prices dynamically contribute incompetence in stock prices in such a way that impact the profits of investors in stock market.

## Introduction

In modern era crude oil is recognized an essential factor to manufacture any product in any economy, crude oil prices variations can affect the economy growth and development either in positive or negative way. Vo [1] stated that rise in oil prices causes to raise the production costs that causes to raise the inflation rate and high inflation rate in economy adversely affect the economic growth. Noor and Dutta [2] stated that oil prices is important element for an economy, variations and instability in oil price can adversely influence the share prices in stock markets. Ciner [3] stated that prices of oil can impact the stock returns either in positive or negative ways. Oil price causes to affect the examined cash flows by their consequence on the economic growth. The prices of oil can disturb the discount rate that is applied to worth the shares in the stock exchange by variation in inflation. Bouri [4] stated that raise in the prices of oil causes to raise the prices of the equity markets share that adversely affect the income of the investors which lead to instability in the financial markets and the economic activities in any economy. Mohanty et al. [5] stated that the raise in the prices of oil affect the company share prices positively or negatively. They stated that raise in the oil prices positively affect the revenue of crude oil manufacturing companies and this raise in the prices of oil causes to raise the income of the oil productions companies. On the other side Phan et al. [6] argued that raise in prices oil is very expensive for the enterprises; because oil is used for production of products. Whenever the price of the crude oil increases so it directly affects the consumer’s purchasing power in economy, raise in prices of oil can be in shape of petroleum products prices.

Crises in prices of oil in 1970s cause the developed economies to faces the problem of recession in their economies. To solve the problem of recession different researchers have scrutinized the influence of variations in the oil prices on different economic factors. These researches for example included, i.e. Cunado and Gracia [7] scrutinized the influence of oil prices on the economic growth of different countries. The examined results indicated that the prices of oil Granger-causes the economy development. Jin [8] scrutinized the influence of oil prices on economic growth. They stated that the raises in the prices of oil have negative impact on the economic development in Japan and China. Rafiq et al. [9] studied the association of oil prices with different economic factors and they stated that the oil price raise has an essential effect on the unemployment rate and the investment rate in Thailand. Du et al. [10] indicated that oil have an important influence on the economic development and inflation rate in China. Fan et al. [11] stated that the oil prices uncertainty affects the economy of China in the short run. Papapetrou [12]; Bahmani-Oskooee et al. ([13] and Bahmani-Oskooee [14]) stated that normally shock in the prices of oil impact negatively the economic development in Greece and Turkey. Cunado and Gracia [15] revealed that raise in oil prices effect the share prices negatively in twelve countries of Europe that import oil from other countries. Nusair [16] stated raise in the oil prices have positive influence the productions output in GCC countries and the similar effect of oil prices are pointed out Iranian economy by Dazaji [17]. Allegret et al. [18] pointed out that increase in the oil prices positively affect the current account balances of twenty seven oil export countries. Overall, the early researches stated that increase in the prices of oil impact differently the countries that import and export. Wang [19] stated that the raise in the prices of oil impact on the personal consumption expenditures higher as compared to decrease in prices of oil.

Early studies use linear models to check the association between oil prices and stock returns. This study scrutinized the nonlinear impact of crude oil prices on Shanghai stock exchange returns. This is first research in China to scrutinize the asymmetric association between oil prices and the stock returns by applying monthly and weekly time series data from January 2000 to December 2018 to examine the asymmetric association between the study variables by utilizing nonlinear ARDL model. Non-linear ARDL model have different advantages as compared to other traditional cointegration model. Asymmetric ARDL model is used when the series of variables are I(0) or I(1) or both of them. To apply nonlinear ARDL model it is compulsory to examine that any variables are not stationary at I(2), otherwise results of the asymmetric ARDL model will be misleading. In nonlinear ARDL model we can use different lag order for variables while the other cointegration method needs the same order of lag. Taking lag of variables helps to remove the problem of endogeneity. Nonlinear ARDL model can be applied for small samples Pesaran and Pesaran [20]; Pesaran et al. [21] and Shin et al. [22].

## Literature review

Jones and Kaul [23] scrutinized the impact of prices of oil on different countries shares market returns; they stated that the prices of oil adverse impact on the share returns. Sadorsky [24] stated that the prices of oil play very essential role in the economy growth; Vector auto regression was applied to scrutinize association of oil prices with the stock returns. The examined outcomes indicated that prices of oil have negative influence on the share returns. Hammoudeh and Li [25] stated raise in oil prices having significant effect on stock returns in countries that export oil to other countries. Basher and Sadorsky [26] argued that oil prices in emerging countries adverse impact on share prices of emerging countries. Nandha and Faff [27] scrutinized association of oil prices with share returns in thirty five different index and they pointed out that raise prices of oil adversely affect the returns of share in these indexes. Miller and Ratti [28] examined the association of oil prices with the share returns in different countries. The examined outcome indicated that raises in the prices of oil have an adverse relationship with share returns in different countries stock exchange. Chen [29] inspected the influence of high prices of oil on the share returns in stock exchange; examined outcome indicated that increases oil prices causes variations in emerging stock market’s returns. Lee and Zeng [30] scrutinized the asymmetric association of oil prices with stock returns in G7 countries stock exchange. They stated prices of oil have dissimilar effect share returns. Basher and Haug [31] studied association of oil prices with stock returns and the exchange rate in emerging countries; they stated increase in prices of oil have negative association with returns of stock in emerging stock exchanges. El-Sharif et al. [32] scrutinized relationship of stock returns with oil prices in London stock exchange; examined outcomes showed raise in the prices of oil positively impact stock returns.

Kilian and Park [33] examined the influence of oil prices on stock returns, examined outcomes shows 22% variation in stock returns is caused due to raise in the prices of oil in the USA. Narayan and Narayan [34] scrutinized asymmetric influence of oil prices on stock returns. They stated that raise and the decline in the prices of oil are very important estimators of the stock returns, further that stated that decline prices of oil having great impact on the returns of stock as compared to raises in oil prices. Arouri and Rault [35] examined the link of stock returns with prices of oil. They stated positive relationship prices of oil and stock returns while the results of SUR model indicated raises in oil prices positive impact on stock market returns. Henriques and Sadorsky [36] studied association between stock returns with different variables. They stated that prices of oil have association with stock returns. Apergis and Miller [37] investigated that influence of prices of oil shocks on stock returns in international stock market. They stated that shock in prices of oil does not impact the stock returns in international stock markets. Narayan and Sharma [38]; Moya-Martínez et al. [39] scrutinized the association of stock returns with prices of oil. They stated that stock returns are influenced by oil prices significantly.

Ajmi et al.[40] scrutinized the influence of oil prices on stock returns in MENA different countries; they checked the nonlinear influence of oil prices on the stock returns; they pointed out crude oil have a non-linear influence on stock in MENA countries. Alsalman and Herrera [41] investigated association of oil prices with stock returns; they stated raise in the oil price has irregular belongings with returns of some industries. Narayan and Gupta [42] studied the positive and negative shock on the stock returns and they pointed out that the fall in the prices of oil more precisely estimate the stock returns as compared to growth in oil prices in United States of America. Ghosh and Kanjilal [43] inspected nonlinear influence of oil prices with stock returns in Indian stock market, they pointed out that nonlinear and significant association exists amongst oil prices and returns. Salisu and Isah [44] scrutinized nonlinear Panel cointegration of oil prices with returns, they stated upsurge in oil price effect asymmetrically the stock returns in oil export and oil import countries. Wen et al. [45] studied risk spillover impact between oil and stock markets by utilizing multivariate quantile model and impulse response function by using daily basis data from January 4, 2000 to August 31, 2018. The examined results stated that asymmetry in spillover impact indicate significantly impact on upside quantile but non-significantly impact on downside quantile.

## Materials and methods

This research utilized monthly and weekly time series data for analysis that is mainly obtained from Federal Reserve Bank of St. Louis https://fred.stlouisfed.org/ and yahoo finance https://finance.yahoo.com/, spanning January 2000 to December 2018, for prices of oil (WTI) and the Shanghai Stock exchange composite index respectively. The used datasets were composed in a legitimate manner, completely obeying with the terms of service of the both sources. By following Qaio et al. [46]; Botta et al. [47]; Ranco et al. [48]; Khan et al. [49] and Yun and Yoon [50] returns of both variables are calculated by taking the natural logarithm difference of stock returns of Shanghai stock exchange composite index and the (WTI) oil prices returns, as shown in the following Equations.

$${SSERM}_{t}=\ln\;\left(\frac{{SSERM}_{t}}{{SSERM}_{t-1}}\right)$$ 1

$${OilpM}_{t}=\ln\;\left(\frac{{OilpM}_{t}}{{OilpM}_{t-1}}\right)$$ 2

Where SSERM and OilpM are the returns of Shanghai stock exchange composite index and the oil prices (WTI) returns respectively, *SSERM*_*t*_ and *OilpM*_*t*_ are the today prices and *SSERM*_*t*−1_, *OilpM*_*t*−1_ are the previous day prices. Following is the basic linear model for returns of Shanghai stock exchange composite index and the oil prices (WTI).

$${SSERM}_{t}=\beta_{0}+\beta_{1}{OilpM}_{t}+\varepsilon_{t}$$ 3

In the above equations β_0_ is the constant, ε_t_ is the error term, SSERM is the returns of Shanghai stock exchange composite index. Shanghai stock exchange returns is applied as a dependent variable while Oilpm is the prices of oil (WTI). Monthly and weekly time series data were used for both variables from January 2000 to December 2018.

Early researches used only linear models for checking the relationship of stock returns with oil prices. This research used non-linear autoregressive distributed lag model for checking the asymmetric long run and the short run association between the series. The purpose of this research is to scrutinize the non-linear associations between stock returns of shanghai stock exchange composite index and the (WTI) oil prices, Asymmetric form of the model is the following Pesaran et al. [21] and Shin et al. [22].

$${SSERM}_{t}=\beta_{0}+\beta_{1}^{+}{OilpM}_{t}+\beta_{2}^{-}{OilpM}_{t}+\varepsilon_{t}$$ 4

Where β_0_ is the constant coefficient of the estimation, β indicate the long-run coefficients of the independent variables, and ε_t_ is the examined error term for t = 1, 2…n (n = 216).

## Asymmetric autoregressive distributed lag

In order to check asymmetry relationship among a set of variables, the nonlinear autoregressive distributed lag model can be applied. Asymmetric ARDL was developed by Shin et al. [22] for checking the positive and negative influence of the independent variables on the dependent variable in long run and short run. Non-linear ARDL is the asymmetric form of the linear ARDL model of Pesaran et al. [21]. The uniqueness between ARDL and NARDL model is that the linear ARDL model does not study the option of negative and positive variations of the independent variables that have different effect on the dependent variable. The NARDL model not only permits to identify the presence of nonlinear relationship that independent variables may have on the dependent variable, but NARDL model also allows for checking cointegration in a single equation framework. NARDL model have some advantages as compared to other cointegration model this is applied mostly for time series data, such as its elasticity about the order of integration of the variables involved, stationarity is not problem for NARDL model but it is required to check that any variable is not stationary at I(2) stated by Pesaran and Pesaran [20] and Ibrahim[51], the option of checking the hidden cointegration among the dependent and independent variables, avoiding to ignore any association which is not noticeable in a conventional linear model and a better performance in small samples. To assist the interpretation of the NARDL model, we first used the expression of the linear (ARDL) model given by the following model.

$${\Delta SSERM}_{t}=\beta_{0}+\beta_{1}\sum_{t=1}^{t}\Delta {SSERM}_{t-1}+\delta_{2}{SSERM}_{t-1}+\delta_{3}{Oilpm}_{t-1}+\varepsilon_{t}$$ 5

In the above equations SSERM is the monthly and weekly stock return of Shanghai stock exchange composite index, Oilpm is the oil prices (WTI), constant term is β0, β1 is the error correction in the short run, δ_2_ and δ_3_ are the long term relationship and εt is the error term. Wald test (F-statistics) is used to choose that cointegration exist among the study variables or not. Based on the above equation, F-test is applied and the result is compared to the bounds upper and lower critical values supplied by Narayan [52]. Based on the results of F-statistics three possible conclusions can be decided. Firstly if calculated F-statistics value is less than the I(0), so cointegration did not exists among the study variables; secondly if the calculated F-statistic value is higher than the I(I) so cointegration exists among the study variables. Lastly if the calculated F-statistics value between the I(I) and I(0) so it is inconclusive. Appropriate lags assigned by Akaike Information Criteria (AIC). If the Bounds test suggests cointegration, the following step is to calculate the long run equilibrium and restricted ECM. From this estimation, the short-run dynamic effects, and the long-run equilibrating association between the variables can be measured.

$${\Delta SSERM}_{t}=\beta_{0}+\beta_{1}\sum_{t=1}^{t}\Delta {SSERM}_{t-1}+\beta_{2}\sum_{t=1}^{t}\Delta {Oilpm}_{t-1}+\delta_{2}{ECT}_{t-1}+\varepsilon_{t}$$ 6

(ECT_t−1_) is the error correction term obtained from the cointegration model. Error correction term coefficient range -1 to 0 and significantly negative. Error correction term indicates the speed of adjustment such that when the dependent variable exceeds the long-run relationship with the independent variable, they adjust downwards at a rate within the range.

Based on Eqs 3 and 4 the following asymmetric models are based.

$${\Delta SSERM}_{t}=\beta_{0}+\beta_{1}\sum_{t=1}^{t}\Delta {SSERM}_{t-1}+\delta_{1}{SSERM}_{t-1}+\delta_{2}^{+}{Oilpm}_{t-1}+\delta_{2}^{-}{Oilpm}_{t-1}+\varepsilon_{t}$$ 7

$${\Delta SSERM}_{t}=\beta_{0}+{\beta_{1}\sum_{t=1}^{t}\Delta {SSERM}_{t-1}+\beta }_{2}\sum_{t=1}^{t}\Delta Oilpm_{t-1}^{+}+\beta_{3}\sum_{t=1}^{t}\Delta Oilpm_{t-1}^{-}+\delta_{1}{ECT}_{t-1}+\varepsilon_{t}$$ 8

In the above Eqs 7 and 8, $\delta_{2}^{+}{Oilpm}_{t-1}+\delta_{3}^{-}{Oilpm}_{t-1}$ and $\beta_{2}\sum_{t=1}^{t}\Delta Oilpm_{t-1}^{+}+\beta_{3}\sum_{t=1}^{t}\Delta Oilpm_{t-1}^{-}$ are the positive and the negative changes in oil prices respectively that affect the stock returns.

$${Oilpm}^{+}=\sum_{t=1}^{t}\Delta Oilpm_{t-1}^{+}=\sum_{t=1}^{t}max(\Delta {Oilpm}_{t},0)$$ 9

$${Oilpm}^{-}=\sum_{t=1}^{t}\Delta Oilpm_{t-1}^{-}=\sum_{t=1}^{t}min(\Delta {Oilpm}_{t},0)$$ 10

For checking the stationarity of the variables we applied two most common unit root tests i.e. ADF and PP tests Asteriou and Hall [53]. Different diagnostic tests such as such as normality of residuals was checked by applying the serial correlation was checked by applying the Breusch–Godfrey serial correlation LM test and the heteroskedasticity was checked by applying the Breusch–Pagan–Godfrey. Stability of the coefficients is checked through the Cumulative sum (CUSUM) and the cumulative sum of square (CUSUMSQ) tests respectively.

## Results and discussion

Table 1 indicates the results of unit root test. ADF test and PP test were utilized to examine the stationarity of variables. Stationarity of variable is essential to be check before applying ARDL model that any series is not stationary at I(2) otherwise the outcomes will not be correct. Ouattara [48] stated that the ARDL results will be incorrect if any of the series are stationary at I(2). Examined outcomes of ADF and PP specify oil prices and the stock returns are stationary at I(0) and I(I). Both tests results showed that ARDL bounds test can be applied because any variables are not stationary at 1(2).

**Table 1:** ADF and PP unit root test.

	ADF		PP	
Level				
Variables	Intercept	Trend & Intercept	Intercept	Trend & Intercept
OILPM	-12.0099***	-12.0301***	-11.9831***	-11.9998***
SSERM	-13.6512***	-13.5358***	-14.0580***	-14.0361***
First Difference				
OILPM	-8.7685***	-8.7433***	-83.3672***	84.8911***
SSERM	-12.9474***	-12.9181***	-62.7302***	-62.5093***

*** p < .001

Table 2 shows the results of lag criteria for choosing lags for nonlinear ARDL model. For non-linear ARDL model different lags can be utilized for dependent and independent variables. It is necessary to select suitable lag for dependent and independent variables before applying nonlinear ARDL model. AIC and SBC are two well-known lag selection approaches in time series data and mostly applied for lag selection. In this research paper we used AIC method for lag selection for nonlinear ARDL model. The results of AIC indicate that lag two is the most suitable lag for nonlinear ARDL model.

**Table 2:** VAR Lag order selection criteria.

Lag	LogL	LR	FPE	AIC	SC	HQ
0	833.9566	NA	1.78e-06	-7.5632	-7.5323	-7.5507
1	845.6269	23.0222	1.66e-06	-7.6329	-7.5404*	-7.5955
2	853.4314	15.2542	1.60e-06*	-7.6675*	-7.5133	-7.6052*
3	854.0825	1.2607	1.65e-06	-7.6371	-7.4211	-7.5499
4	860.1755	11.6874	1.62e-06	-7.6561	-7.3784	-7.5440
5	861.4453	2.4125	1.66e-06	-7.6313	-7.2919	-7.4943
6	867.5688	11.5234*	1.63e-06	-7.6506	-7.2495	-7.4886
7	871.7120	7.7213	1.63e-06	-7.6519	-7.1891	-7.4650
8	871.8405	0.2371	1.69e-06	-7.6167	-7.0922	-7.4049

* indicates lag order selected by the criterion.

Table 3 indicates asymmetric ARDL bound testing approach results. Bound testing method is applied for the long run association. The above table indicates results of asymmetric bound testing approach at 5% level of significance. According to Table 4 the critical value of asymmetric bound testing approach at 5% the I(0) value is 3.79 and the I(I) value is 4.85 respectively, while the calculated F-statistic value is 14.2494 and 74.3078 for monthly and weekly respectively that are higher as compared to I(I) value so it indicates that asymmetric cointegration exist.

**Table 3:** Asymmetric ARDL Bounds test.

Monthly			Weekly		
Test Statistic	Value	K	Test Statistic	Value	K
F-statistics	14.2494	2	F-statistics	74.3078	2

**Table 4:** Critical Bounds value.

Critical Value Bounds		
Significance	I(0)	I(I)
10%	3.17	4.14
5%	3.79	4.85
2.5%	4.41	5.52
1%	5.15	6.36

Table 5 indicates the results of asymmetric ARDL long run coefficients. Stock returns are used as dependent variable while oil prices are used independent variable in this research paper. The examined results of asymmetric ARDL indicate that when the oil prices increase (positive change prices of oil) negatively affect the stock returns while when the prices of oil decreases (negative change in prices of oil) positively affect the stock returns. Examined results of positive change in prices of oil indicate that a 1% rise in prices of oil negatively affect the stock returns in Shanghai stock exchange that causes to decrease the stock returns about 41.39%. Positive shocks in oil prices impact adversely the stock returns because with raise in oil prices, causes to increase the daily use product prices because of high production cost and the investment ability of a normal investor will decrease because of low saving rate. Weekly results indicate symmetric impact on stock returns; both increase and decrease in weekly oil prices negatively impact the stock returns. Examined results of negative change in oil prices indicate that a 1% decrease in prices of oil positively impact the stock returns in Shanghai stock exchange increases up to 56.46%. This indicates that with decrease in oil prices the production cost of different product decreases and the saving of a normal investor increases. The demand of different share in stock market increase and the returns increases. Our results of asymmetric ARDL are same as previous researchers. Ajmi et al. [40] investigated nonlinear relation amid oil prices and stock returns in the MENA. The authors pointed out nonlinear relationship among the study variables. Alsalman and Herrera [41] studied non-linear association of oil prices with the stock returns of different sector. The authors pointed out that raise in prices of oil or fall having asymmetric influence on stock returns in different sectors. Narayan and Gupta [42] scrutinized association amid oil prices and the US stock returns. The authors stated decreases in oil price have positive effect on US stock returns as compared to increase in oil prices, Raise in oil prices adversely influence on US stock returns. Ghosh and Kanjilal [43] studied the nonlinear cointegration association between the prices of oil and the Indian stock exchange returns. They stated nonlinear relation exists amid the oil prices and the stock returns. Salisu and Isah [44] applied nonlinear Panel ARDL technique to scrutinize the short run and the long run association amid oil prices and stock returns; they pointed increase and decrease in prices of oil asymmetrically influence the stock returns.

**Table 5:** Asymmetric ARDL long run coefficients.

Monthly				Weekly		
Variable	Coefficient	Std. Error	Prob.	Coefficient	Std. Error	Prob.
OILPM_POS	-0.4139	0.1830	0.0248	-0.0029	0.0039	0.4639
OILPM_NEG	0.5646	0.2628	0.0204	-0.0026	0.0024	0.5241
C	0.0052	0.0117	0.6562	1.0009	0.0019	0.0000

Table 6 indicates the result of different diagnostics statistics. The adjusted R squares values indicate that 51% and 44% variation in monthly and weekly stock returns respectively are caused by oil prices. AIC and SIC value is applied to choose the suitable lag for series. According to rule of thumb we select the minimum value for lag selection. The examined value of AIC is the minimum that is used for lag selection. Durbin Watson statistics value shows that no autocorrelation problem of in our examined model. The P-value of F-statistics is 0.0000 that indicates that the model if fit.

**Table 6:** Diagnostics statistics for asymmetric ARDL long run coefficients.

Adjusted R Square	0.5168	0.4474
Akaike information criterion	-4.0516	-5.1666
Durbin-Watson Statistics	2.0359	1.7025
F-Statistics	30.8228(0.0000)	78.9457(0.0000)

Table 7 indicates the results of short run asymmetric model. Short run asymmetric ARDL model verified non-linear long run ARDL results. The examined results show that raise in oil prices has an adverse and non-significant influence on the stock returns in Shanghai stock exchange. The examined results show that a 1% raise in the oil prices causes to decrease the stock returns about 11% to 13%. While weekly oil prices indicate symmetric impact on the stock returns. Oil prices play very essential role in the financial market. The examined results of short run ARDL is same with previous researchers. Jones and Kaul [23], Sadorsky [54] and Ciner [55] stated that rise in prices of oil causes to decrease the stock returns. Henriques and Sadorsky [36]; Apergis and Miller [37] and Al Janabi et al. [56] stated that rise in oil prices have non-significant influence on stock returns. Basher et al. [31] stated that rise in prices of oil react to decrease the stock prices in emerging market. The examined results show that decrease in oil prices has supportive and significant influence on stock returns in Shanghai stock exchange. One percent decrease in prices of oil causes to raise the stock returns about 25%. Our results are same with Ajmi et al. [40], Afshar et al. [57]; Hatemi-J et al. [58] and Huang et al. [59] stated that oil prices have non-linear and significant influence on stock returns. Phan et al. [6] studied the asymmetric association of oil prices and the stock. They pointed out that raise and fall in oil prices asymmetrically influence the stock returns. Kisswani and Elian [60] investigated asymmetric relations of stock returns and the oil prices. Based on the examined outcomes of oil prices; oil prices adversely influence the stock returns in long-run. Raza et al. [61] investigated the asymmetric association between oil prices, they pointed out prices of oil adversely influence the stock returns. Error correction term indicates the speed of correction from previous disequilibrium. ECT values of both monthly and weekly are negative and statistically significant as expected. ECT of monthly and weekly results indicate that 77% and 83% correction in previous disequilibrium over time as the variables move toward stable long run relationship.

**Table 7:** Asymmetric ARDL short run coefficients.

Monthly				Weekly		
Variable	Coefficient	Std. Error	Prob.	Coefficient	Std. Error	Prob.
D(OILPM_POS)	-0.1217	0.0898	0.1766	0.1016	1.7680	0.0774
D(OILPM_POS(-1))	-0.1127	0.0958	0.2405	-0.0019	-0.3440	0.9728
D(OILPM_POS(-2))	-0.1339	0.0798	0.0948	-0.0431	-0.7543	0.4508
D(OILPM_NEG)	0.1061	0.0883	0.2309	-0.0422	-0.8871	0.7142
D(OILPM_NEG(-1))	0.2527	0.1081	0.0203	-0.0022	-0.6371	0.5242
ECT(-1)	-0.7701	-0.8363	0.0000	-0.8383	-14.9251	0.0000

Table 8 indicates results of different diagnostic statistics. Ramsey reset test is used to check that the model is specified correctly or not. Results of Ramsey reset test indicate that the model is properly quantified. LM and Breusch–Pagan–Godfrey are utilized to scrutinize the serial association and heteroscedasticity respectively. The examined P-value of X^2^ are 0.2179 and 0.2402 respectively that indicates that examined model is free from the problem of serial association and heteroscedasticity.

**Table 8:** Diagnostic statistics.

Test	P-Value of X^2^	Decision based on P-Value
Ramsey Reset Test	0.8865	Model is properly specified
LM	0.2179	No Serial Correlation problem
Breusch–Pagan–Godfrey	0.2402	No Heteroscedasticity problem

**Fig 1:**
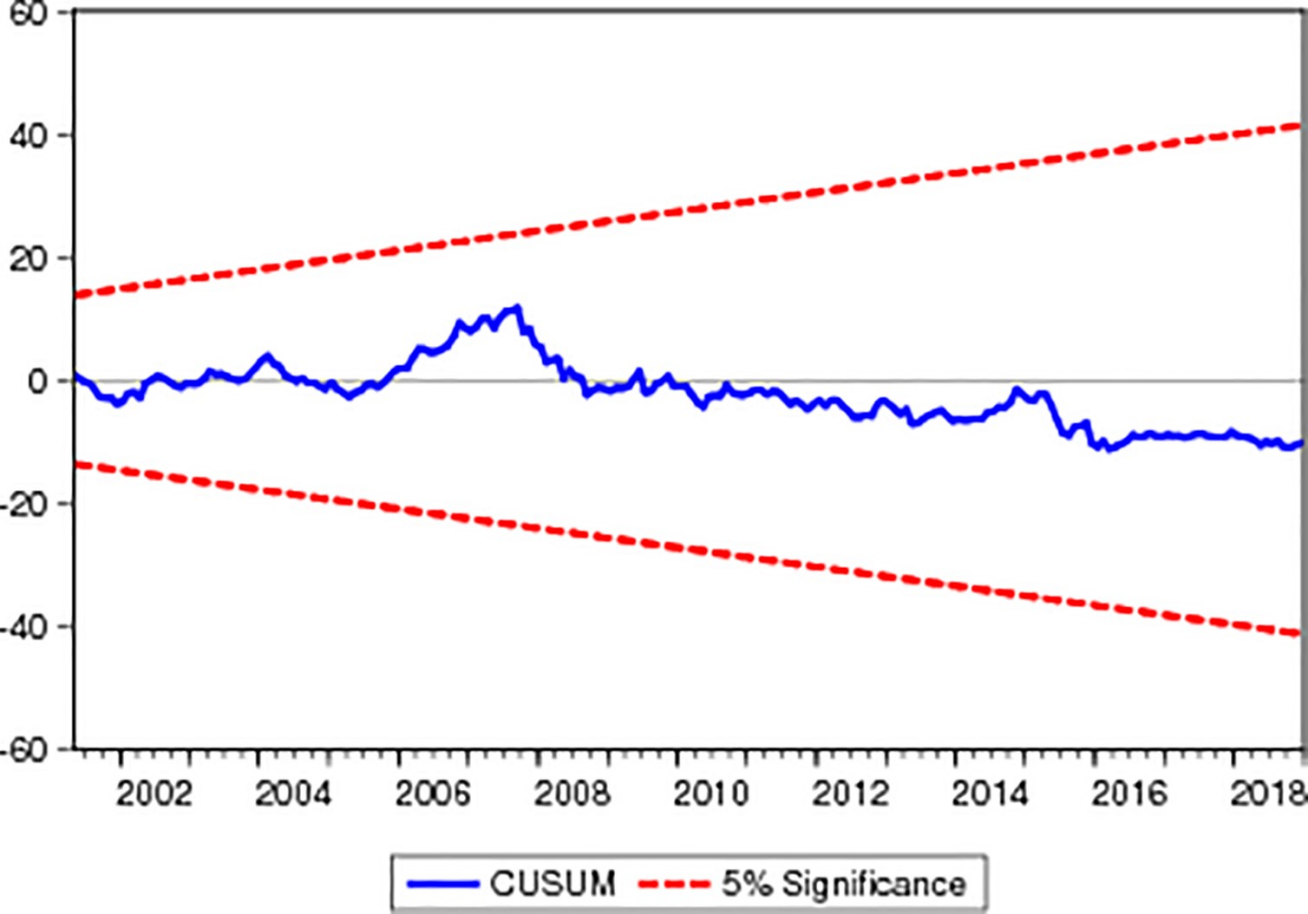
CUSUM.

**Fig 2:**
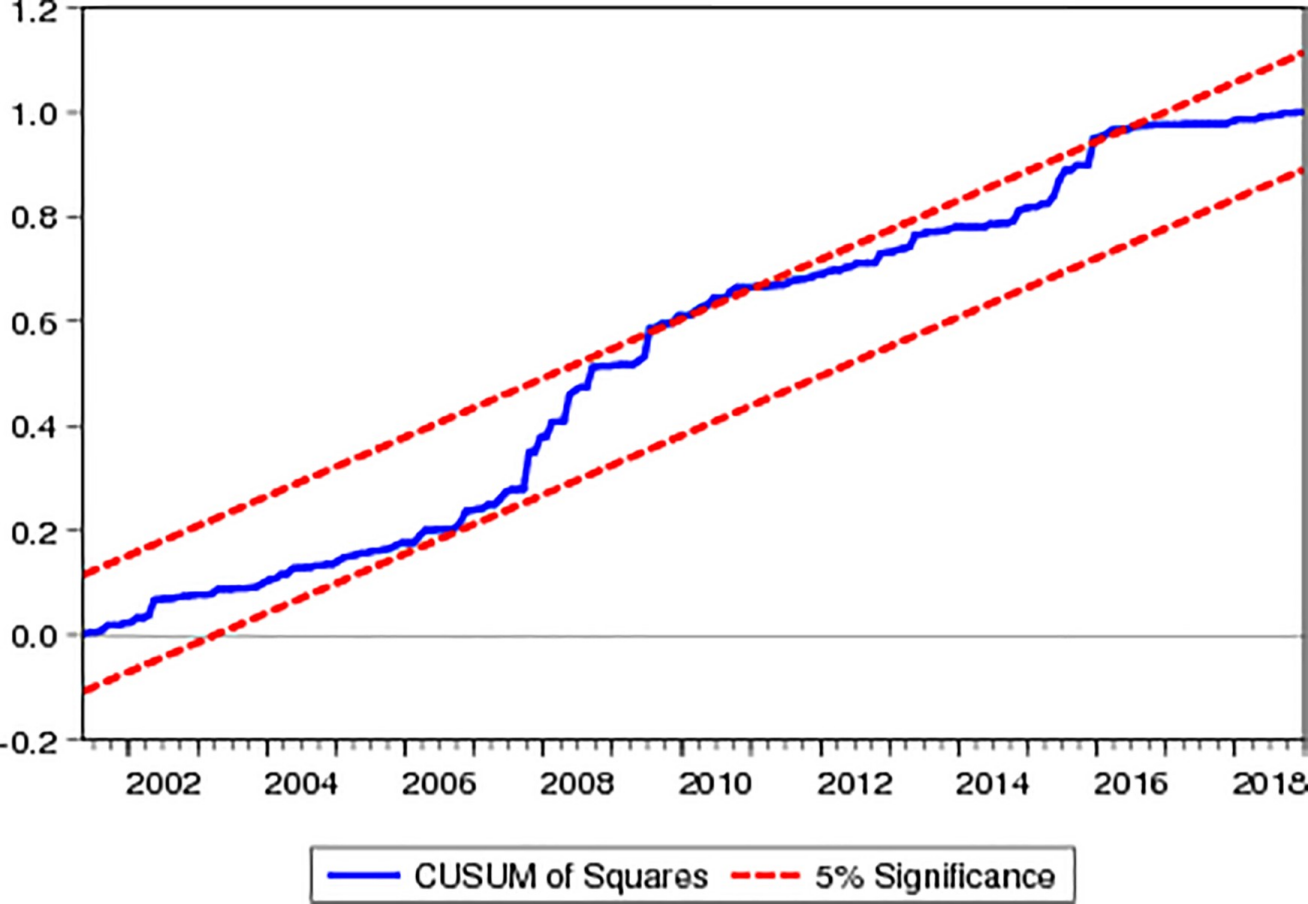
CUSUM of Squares.

Figs 1 and 2 indicate the CUSUM and CUSUM of squares graph respectively; both figures specify that coefficients stable at 5% level of significance because the blue line of the both figure are inside the red lines.

**Fig 3:**
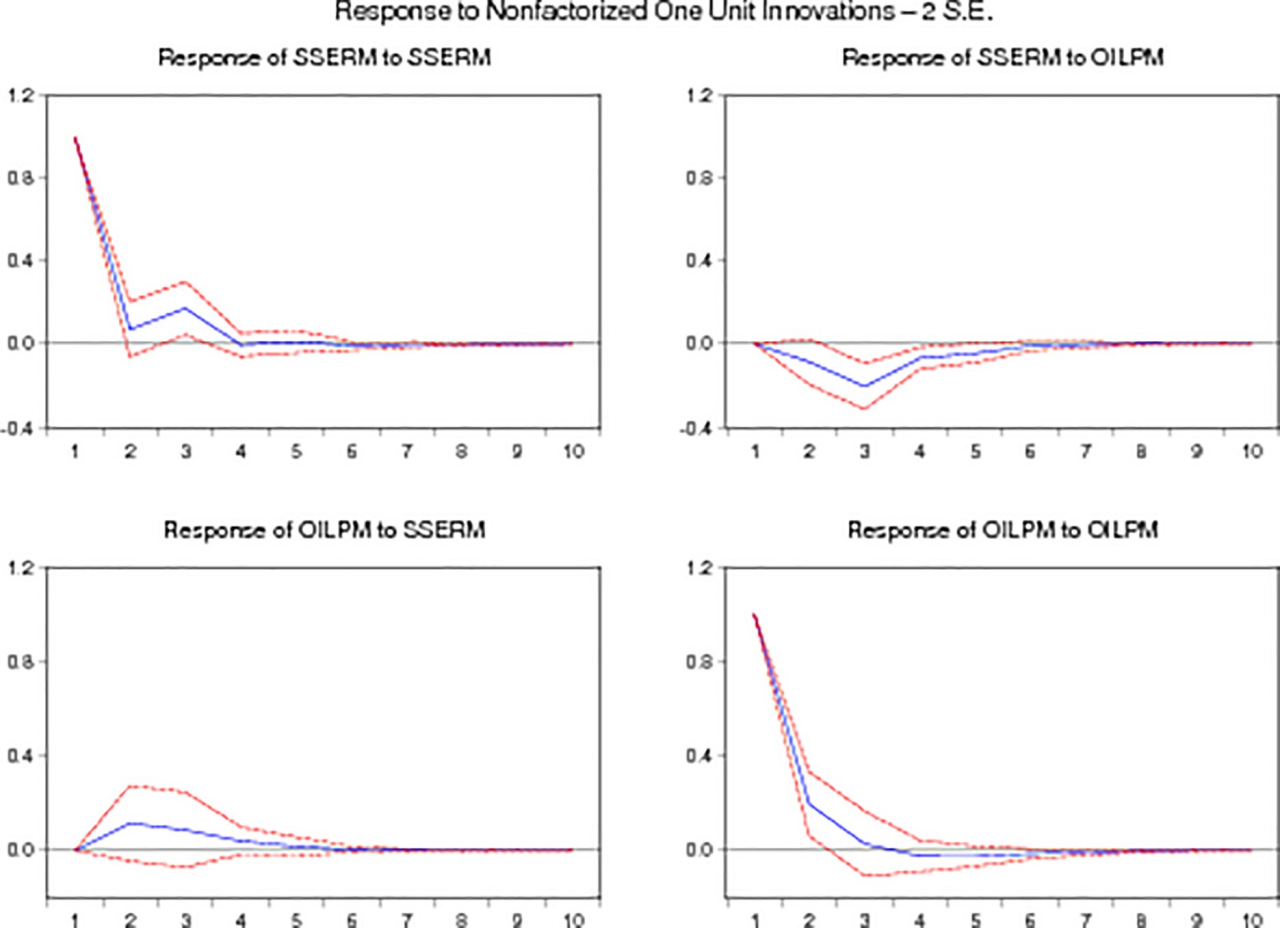
Monthly impulse responses graphs.

**Fig 4:**
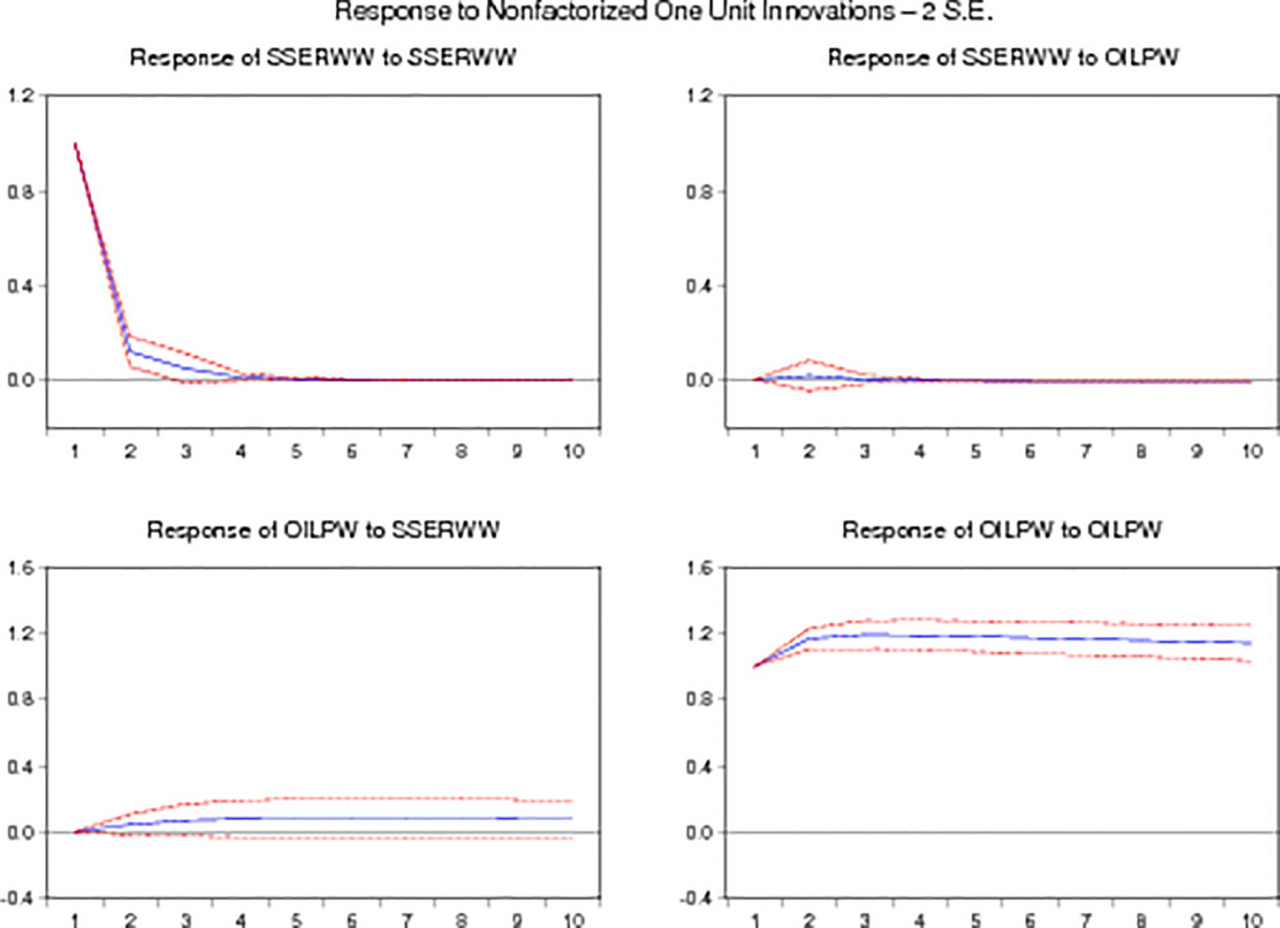
Weekly impulse responses graphs.

Figs 3 and 4 indicate the impulse responses graphs of monthly and weekly. The above graphs show the response of monthly and weekly stock returns of Shanghai stock exchange to change in monthly and weekly oil prices. Monthly graph of impulse response indicate that stock returns have positive response to oil prices in start but it become negative after sometime while the graph of weekly impulse response indicate a positive response of stock returns to oil prices.

## Conclusions and recommendations

This research scrutinize the asymmetric effect of oil prices on stock returns in Shanghai stock exchange by using monthly and weekly time series data from January 2000 to December 2018 for examination. Asymmetric ARDL model were applied to scrutinize the short and the long run association between the study variables. Previously research used linear cointegration model to examine the long run and the short run association, but we study the non-linear long run and short run associations between the study variables, because it necessary to check that what is the influence of raise and fall in oil prices on the stock returns. It is essential to examine the stationarity of each variable that none of the variable is stationary at I(2) before applying asymmetric ARDL model. ADF and PP were applied to check the stationarity of each variable and the examined results confirm that any variable is not stationary at I(2). Asymmetric ARDL is better as compared to other cointegration method because in asymmetric ARDL model different lags for variables can be applied. AIC is used for lag selection because the calculated value of AIC is the minimum as compared to other lag selection criterion. The examined results of the asymmetric long run and short run indicate that when the oil prices increase so it has an adverse influence on the stock returns while a negative change in oil prices have a positive impact on the stock returns. Results of weekly change in oil prices indicate symmetric impact on stock returns, both increase and decrease in weekly oil prices negatively impact the stock returns. Furthermore, it is pointed out that the information apprehended by the changes in oil price can be applied to describe possible asymmetric reaction in the developing countries stock markets. Oil prices play very important role in financial market. The movement of oil prices impacts the behavior of investor that impact the stock prices either in positive or negative way that causes uncertainty on the economy growth. The results indicate that association between oil prices and stock returns in China rely on policies uncertainty. It is necessary for policy makers to organize such strategies that help to reduce the oil shocks harmfulness on financial market. For instance, employing alternative energy resources can decrease the dependence on oil for production purposes. It is very important for investors to know asymmetric association to make appropriate investment decisions because it will qualify investors to decide profit generating stocks at right time to invest. Moreover, investors also need to observe the economic situation and their exposure to the stock market.
